# Emerging mastitis-associated *Corynebacterium parakroppenstedtii* and *Corynebacterium pseudokroppenstedtii*: clinical, microbiological, and epidemiological features from a two-year study in Guangdong, China

**DOI:** 10.3389/fcimb.2025.1723551

**Published:** 2026-01-14

**Authors:** Minling Zheng, Qiongdan Mai, Yasha Luo, Xiaowei Chen, Weiming Lai, Junfei Guo, Yanting Qin, Lingling Tang, Zhiyu Li, Hongyu Li, Wenyu Deng, Pinghua Qu, Mingyong Luo

**Affiliations:** 1 Department of Clinical Laboratory, Guangdong Women and Children Hospital, Guangzhou, China; 2 Department of Laboratory Medicine, Panyu Hospital of Chinese Medicine, Guangzhou, China; 3 Guangzhou Medical University, Guangzhou, China; 4 School of Medicine, Foshan University, Foshan, China; 5 Department of Clinical Laboratory, Women and Children’s Hospital, Southern University of Science and Technology, Guangzhou, China

**Keywords:** *C. parakroppenstedtii*, *C. pseudokroppenstedtii*, epidemiology, mastitis, microbiology

## Abstract

**Background:**

*Corynebacterium parakroppenstedtii*, and *C*. *pseudokroppenstedtii* are emerging as significant pathogens in mastitis. Despite its clinical significance, data on its epidemiological, microbiological, and clinical features remain limited.

**Methods:**

We conducted a comprehensive study on mastitis cases associated with *Corynebacterium kroppenstedtii* complex (CKC) in Guangdong, China (September 2021–September 2023). A total of 101 bacterial isolates were collected and initially identified as CKC using MALDI-TOF MS. Species-level confirmation was achieved through partial sequencing of the 16S rRNA, *ropB*, and *fusA* genes. Unclassified isolates were further characterized by whole genome sequencing (WGS). Clinical information was collected, and antimicrobial susceptibility testing was performed.

**Results:**

Among the 101cases, *C*. *parakroppenstedtii* accounted for 86% of infections, while *C*. *pseudokroppenstedtii* for 12%, and a potential novel species for the remaining 2%. Notably, no *C*. *kroppenstedtii* infections were detected. Granulomatous lobular mastitis (GLM) was the predominant presentation, occurring in 72% of all cases. Comparative analysis revealed that *C*. *pseudokroppenstedtii* infections were associated with higher rates of pus formation and recurrence, whereas *C*. *parakroppenstedtii* infections were more prevalent among parous women. Moreover, *C*. *pseudokroppenstedtii* exhibited higher resistance rates to ceftriaxone (32.15% vs. 20.00%) and ciprofloxacin (75.00% vs. 19.09%) compared with *C*. *parakroppenstedtii*.

**Conclusions:**

These findings challenge the prevailing understanding that *C*. *kroppenstedtii* is the main pathogen in mastitis and underscore the need for species-level identification to guide diagnosis and optimize antibiotic therapy for CKC-related mastitis. These insights are vital for improving clinical management and informing treatment strategies.

## Introduction

The genus *Corynebacterium* was first proposed in 1896 and described as a group of Gram-positive club-shaped non-spore forming bacteria belonging to the family *Corynebacteriaceae* in the order *Corynebacteriales* (Bernard, 2012; Salam et al., 2020; Oren and Garrity, 2021). To date, over 250 species of *Corynebacterium* have been identified (https://www.bacterio.net/). While most of the clinically isolated *Corynebacterium* species are considered to be opportunistic pathogens (Bernard et al., 2002), a few *Corynebacterium* species have been recognized as major pathogens causing human diseases (Coyle and Lipsky, 1990; Sangal and Hoskisson, 2016). Among these, *Corynebacterium kroppenstedtii* (*C*. *kroppenstedtii*), first isolated in 1998 from a pneumonia patient (Collins et al., 1998), is notable for its lipophilic nature, absence of mycolic acids, and preferential colonization of lipid-rich tissues such as the breast (Le Flèche-Matéos et al., 2012; Saraiya and Corpuz, 2019; Roth et al., 2020). The rising prevalence of clinical mastitis cases has highlighted *C*. *kroppenstedtii* as a pivotal contributor to disease recurrence, early onset of pus formation and pathogenesis of mastitis, particularly granulomatous mastitis (Taylor et al., 2003; Tauch et al., 2016; Zeng et al., 2022; Chen et al., 2023).

Recent advances in phylogenetic analysis and molecular identification methods have elucidated the genetic diversity of *C*. *kroppenstedtii* strains, allowing for the differentiation of novel species. This has led to the reclassification of the lipophilic *Corynebacterium* bacteria, previously identified as *C*. *kroppenstedtii* in mastitis, mainly into two novel species with highly similar genetic profiles: *Corynebacterium parakroppenstedtii* (*C*. *parakroppenstedtii*) and *Corynebacterium pseudokroppenstedtii* (*C*. *pseudokroppenstedtii*). These two novel species were first proposed by Luo et al. using a polyphasic taxonomic approach in 2022 (Luo et al., 2022). As with *C*. *kroppenstedtii*, *C*. *parakroppenstedtii* and *C*. *pseudokroppenstedtii* are also slow-growing and lipophilic bacteria those mainly found in mastitis (15). Subsequently, Huang et al. introduced the concept of *Corynebacterium kroppenstedtii* complex (CKC), comprising *C*. *kroppenstedtii*, *C*. *parakroppenstedtii*, and *C*. *pseudokroppenstedtii*, reflect the genetic and phenotypic diversity among these closely related species. This reclassification underscores the importance of accurately identifying these similar strains to aid in clinical diagnosis and effective treatments (Huang et al., 2024).

For the identification for CKC strains, although MALDI-TOF MS is generally regarded as an efficient and cost-effective tool for bacterial identification, and 16S rRNA gene sequencing is considered the gold standard, both methods have known limitations. Previous literature has shown that these methods have difficulties in distinguishing CKC strains due to limited reference databases and the high genetic identity among these organisms (Liang et al., 2024; Xiao and Zhao, 2024; Zeng et al., 2024). Further classification was enabled by techniques, including partial gene sequencing of *rpoB* and *fusA*, as well as whole-genome sequencing (WGS). They have provided valuable insights into the genetic and phenotypic diversity of the novel species related to C. *kroppenstedtii* (Luo et al., 2022; Huang et al., 2024).

However, research on the relationship between these novel species and mastitis is limited, and the pathogenesis of *C*. *parakroppenstedtii* and *C*. *pseudokroppenstedtii* remains unclear in mastitis. While Luo et al. (2022) first associated *C*. *kroppenstedtii*-like species with mastitis in China (15), subsequent studies confirmed *C*. *parakroppenstedtii* as a GM pathogen via secretion of the glycolipid virulence factor “corynekropbactins” (Liu et al., 2024). In contrast, the pathogenicity and etiological characteristics of *C*. *pseudokroppenstedtii*, in mastitis remain poorly understood, likely due to the limited number of documented cases.

According to the limited literature, most studies on CKC-related mastitis mainly focused on the comparative analysis of the clinical features of mastitis with or without infection by lipophilic *Corynebacterium*. Retrospective analyses indicate that CKC-positive patients—particularly those with *C*. *kroppenstedtii* or *C*. *parakroppenstedtii*—exhibit more severe manifestations, including larger masses, sinus formation, elevated inflammatory markers (CRP, WBC), and higher recurrence rates (Zeng et al., 2022; Liang et al., 2024; Zeng et al., 2024). However, to date, no research has been conducted a comprehensive comparative analysis of mastitis caused by *C*. *parakroppenstedtii* versus *C*. *pseudokroppenstedtii*.

The current study builds on the CKC concept by analyzing a larger cohort of isolates (n=101) from mastitis cases. We aim to explore and compare the differences in clinical manifestations, epidemiology and microbiological characteristics of mastitis patients with *C*. *parakroppenstedtii* and *C*. *pseudokroppenstedtii*, respectively. These findings will be essential for developing effective diagnostic and therapeutic strategies for emerging CKC-related mastitis.

## Methodology

### Clinical data and isolates

This retrospective study included 101 patients with mastitis confirmed by microbiological evidence of CKC infection, all of whom were treated at Guangdong Women and Children’s Hospital in Guangdong, China, between September 2021 and September 2023. The patients included in this study were comprised of lactating and non-lactating individuals, including those with concomitant infections. For the drug susceptibility test, 27 CKC isolates from our previous study were added to the test as reference samples (Luo et al., 2022). C. *kroppenstedtii* DSM 44385^T^, *C*. *parakroppenstedtii* MC-26^T^ and *C. pseudokroppenstedtii* MC-17X^T^ were included in the present study as reference type strains. Patient information was collected according to the patient’s electronic medical record, including baseline characteristic data, laboratory testing results, clinical treatment and outcomes. A recurrence of the disease is defined as the re-admission of a patient to the hospital for treatment of the same condition, excluding instances where the patient is readmitted for follow-up after an initial improvement. The purified strains were collected by subculturing on Columbia blood agar plates at 35 °C in a 5% CO_2_ atmosphere and stored as glycerol suspensions (30%, vol/vol) with 5% blood at -80 °C.

### Phenotypic testing and MALDI-TOF MS

The biochemical characterization of all isolates was performed using the *Corynebacterium* ID&AST kit (TDR, China) in accordance with the manufacturer’s instructions. Oxidase and catalase activities were assessed by the methods as described previously (Sasser, 1990). Lipophilic test was observed on Columbia blood agar after 24 hours of incubation at 35°C in a 5% CO_2_ conditions with a drop of Tween 80 added to it (Tauch et al., 2016). The morphological characteristics of these strains were Gram stain. All the isolates were initially identified by matrix-assisted laser desorption ionization time of flight mass spectrometry (MALDI-TOF MS) (Bruker Daltonics, Germany).

### DNA sequencing and phylogenetic analysis

Genomic DNAs was extracted from samples using a Bacterial Genomic DNA Rapid Extraction Kit (Sangon Biotech, China) following the manufacturer’s instructions. All clinical isolates were further confirmed by DNA sequencing with 16S rRNA, *rpoB*, and *fusA* genes. PCR amplifications of the above three gene sequences were performed with the corresponding primers as previously described (Weisburg et al., 1991; Khamis et al., 2004; Busse et al., 2019). The quality for all sequences was determined by Chromas (version 1.62) software. The sequences were assembled using DNAMAN (version 7) software and analyzed on the NCBI BLAST website (https://blast.ncbi.nlm.nih.gov/Blast.cgi). Phylogenetic dendrograms were generated using neighbor-joining method with MEGA (version 7) software (Kumar et al., 2016).

For partial isolates that could not be distinguished by 16S rRNA, *rpoB*, and *fusA* gene sequencing, whole genome sequencing was used to further identify and distinguish them. The genomic DNA for genome sequencing was extracted using Bacterial Genomic DNA Rapid Extraction Kit (Sangon Biotech, China) and sequenced on Illumina NovaSeq. The sequence was trimmed by Trim Galore v0.6.0 (http://www.bioinformatics.babraham.ac.uk/projects/trim_galore/). The clean data was assembled and integrated as previously described (Luo et al., 2022). The final genome sequences were annotated by Prokka software (Seemann, 2014). Single nucleotide polymorphisms (SNPs) were detected among the genomes and extracted using SNP-sites (Page et al., 2016). A maximum likelihood phylogeny was constructed from the cgSNPs using the RAxML algorithm (Togkousidis et al., 2023). The visualization and annotation were performed with the tvBOT tool (Version 2.6) (Xie et al., 2023). The average nucleotide identity (ANI) percentage was determined using JSpecies (http://jspecies.ribohost.com/jspeciesws/) and visualized at tvBOT tool.

### *In vitro* antimicrobial susceptibility testing

Antibiotic susceptibility testing was performed by broth microdilution using *Corynebacterium* ID&AST kit (TDR, China) according to the manufacturer’s instructions. Thirteen antimicrobial agents were included: penicillin, ceftriaxone, cefepime, meropenem, vancomycin, gentamicin, erythromycin, daptomycin, tetracycline, trimethoprim-sulfamethoxazole, ciprofloxacin, clindamycin, and linezolid. Susceptibility criteria were interpreted according to the Clinical and Laboratory Standards Institute (CLSI) document M45-A3 (Jorgensen and Clinical, Laboratory Standards I, 2006). Antimicrobial susceptibilities of rifampicin were used Kirby-Bauer test (Oxoid, UK), reference to EUCAST 2025.

### Prediction of antibiotic resistance genes from the draft genome sequencing data

The resistance genes were aligned to the Comprehensive Antibiotic Resistance Database (CARD) and analyzed using Abricate (https://github.com/tseemann/ABRICATE) with minimum thresholds of 90% sequence identity and 90% gene coverage (https://github.com/tseemann/ABRICATE).

### Statistical analysis

In this study, we calculated and displayed the frequency and percentages for categorical variables, while continuous variables were demonstrated as medians and ranges.

## Results

### Sampling and MS identification of CKC-like isolates

A total of 101 CKC-like isolates were obtained from breast specimens of 101 female individuals with mastitis between 2021 and 2023. Among them, 74 isolates were collected from patients diagnosed with granulomatous lobular mastitis (GLM), while 26 isolates were collected from patients diagnosed with mastitis (M). Additionally, one isolate was from a patient diagnosed with plasma cell mastitis (PCM). More details are listed in the **Supplementary Table S1**.

All isolates were first identified by a MALDI-TOF MS using a Bruker Biotyper system. Most (64.5%) of the isolates were reliably identified at the genus level of *Corynebacterium* (score ≥1.7), indicating the limitations of this method for identifying CKC. Specifically, *C*. *kroppenstedtii-*CCUG 44504 and -CCUG 61180 were found to be the predominate species. MS results indicated that the isolates belong to the species *C*. *kroppenstedtii*. The detailed MS results are shown in **Supplementary Table S2**.

### Genotypic identification

**Figure 1:**
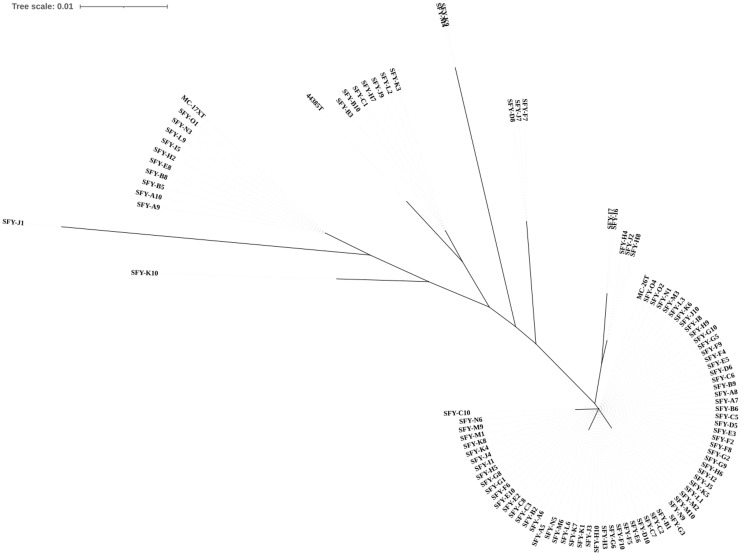
Neighbor-joining tree based on partial *rpoB* gene showing the phylogenetic relationship of 101 CKC isolates and the most closely related species in the genus *Corynebacterium*. Bootstrap values based on 1,000 calculations are shown. The scale bar depicts 0.010 substitutions per nucleotide position.

To further identify the genotype of these isolates and obtain their subspecies information, segments of partial 16S rRNA, *rpoB*, and *fusA* genes were sequenced. Comparative sequence analysis was then performed against the type strains *C*. *kroppenstedtii* DSM44385^T^, *C*. *parakroppenstedtii* MC-26^T^, and *C*. *pseudokroppenstedtii* MC-17X^T^ (**Supplementary Table S2**). As a result, the alignment of partial 16S rRNA gene was unable to differentiate the strains due to an identity greater than 98.5% among all isolates (Yarza et al., 2008). It is expected because previous studies have demonstrated that 16S rRNA is nearly identical among *C*. *kroppenstedtii*, *C. parakroppenstedtii*, and *C*. *pseudokroppenstedtii* (Luo et al., 2022). Accurate identification of species was further determined by the alignment of partial *rpoB* and *fusA* gene. As shown in **Figure 1**, the neighbor-joining tree derived from partial *rpoB* gene sequences reveals that the majority of the 101 isolates are closely related. Among 13 genotypic sequencing isolates, SFY-M4 and SFY-K9 formed a cluster distinct from other reference strains. Another cluster was observed among SFY-D8, SFY-J7, and SFY-F7. SFY-K10 exhibited a unique positioning, distinct from all other groups. The remaining seven isolates (SFY-K3, SFY-L2, SFY-J9, SFY-H7, SFY-C1, SFY-B10, and SFY-B3) demonstrated a coherent relationship with the type strain C. *kroppenstedtii* DSM44385^T^, indicating a close genetic identity. Consequently, 88 out of 101 isolates were successfully classified into the *C*. *pseudokroppenstedtii* and *C*. *parakroppenstedtii* groups, with high identity of *rpoB* of ≥96.6% and ≤98%, respectively (Khamis et al., 2004).

The remaining 13 isolates (SFY-K9, M4, B3, B10, C1, D8, F7, H7, J7, J9, K3, K10, and L2), did not meet the threshold range of partial *rpoB* or *fusA* gene alignment and thus required other identification methods to further confirm their phylogenic information. Therefore, these isolates were subjected to the WGS whose G+C contents ranged from 56.0% to 57.0% and SFY-K9 and M4 both had a G+C content of 56.1%. WGS results showed that SFY-K9 and SFY-M4 had similar genomic size of 2.4 Mb. In contrast, SFY-K10 had a genomic size of 2.5 Mb, SFY-H7 had 2.8 Mb, and the remaining 9 isolates had a genomic size of 2.6 Mb. As demonstrated in **Table 1**, dDDH and ANIb values were utilized to assess their genomic correlation and identify their closest type strain. Besides, pairwise comparisons of ANIbs were conducted between these 13 isolates and their most closely related type strains. As a result, 10 isolates (SFY-B3, B10, C1, D8, F7, H7, J7, J9, K3, L2) were found to be closely related (dDDH > 80%) to each other and shared over 97% identity to with the type strain *C*. *parakroppenstedtii* MC-26^T^, whereas SFY-K10 had an ANIb value of 95.7% with the type strain *C*. *pseudokroppenstedtii* MC-17X^T^. The identity pattern was observed consistently in pairwise comparisons, suggesting that these isolates belong to the *C*. *parakroppenstedtii* and *C*. *pseudokroppenstedtii* groups, respectively. Notably, the two remaining isolates, SFY-K9 and SFY-M4, showed weak genomic identity with MC-17X^T^, MC-26^T^, and DSM 44385^T^. Specifically, the dDDH and ANI values between the type strains and both SFY-K9 and SFY-M4 were all below 45% and 92%, respectively, which are well below the species definition thresholds of 70% for dDDH and 95% for ANI (Goris et al., 2007; Palmer et al., 2020). However, they showed high ANI percentage with P1_C1 and FDAARGOS_1193, suggesting that they may represent the novel species distinct from the three type strains described above. As such, they are classified into a different CKC group in this study.

**Table 1:** Comparative genomic analysis and genomic characteristics of some *Corynebacterium kroppenstedtii* complex isolates and their most closely type strains.

Strain	Pairwise comparison result						Genome characteristics			GenBank ID
	*C*. *parakroppenstedtii.* MC-26^T^		*C. pseudokroppenstedtii* MC-17X^T^		*C*. *kroppenstedtii DSM* 44385^T^		No. of contigs	Size (Mb)	G+C (%)	
	dDDH (%)	ANIb (%)	dDDH (%)	ANIb (%)	dDDH (%)	ANIb (%)				
SFY_K9	44.90	91.75	36.80	89.17	36.90	89.18	4	2.4	56.1	JAWVBD000000000
SFY_M4	44.90	91.78	36.80	89.22	37.00	89.16	3	2.4	56.1	JAWUDO000000000
SFY_B3	91.80	99.03	45.70	91.93	47.60	92.37	70	2.6	56.6	JBBJYY000000000
SFY_B10	91.90	99.04	45.90	91.98	47.60	92.31	49	2.6	56.8	JBBJYZ000000000
SFY_C1	91.90	99.00	46.10	91.96	47.60	92.28	59	2.6	56.8	JBBJZA000000000
SFY_D8	81.00	97.75	46.30	92.00	47.50	92.34	62	2.6	56.7	JBBJZB000000000
SFY_F7	81.00	97.77	46.30	92.03	47.50	92.39	42	2.6	56.7	JBBJZC000000000
SFY_H7	95.90	99.03	46.00	91.84	47.70	92.22	391	2.8	56.8	JBBJZD000000000
SFY_J7	80.90	97.71	46.30	92.01	47.50	92.36	72	2.6	56.8	JBBJZE000000000
SFY_J9	91.80	99.03	45.90	91.94	47.60	92.34	54	2.6	56.8	JBBJZF000000000
SFY_K3	100.00	99.99	45.90	91.96	47.60	92.36	45	2.6	56.7	JBBJZG000000000
SFY_K10	44.70	91.57	60.10	95.70	47.80	92.36	55	2.5	57.0	JBBEGR000000000
SFY_L2	91.90	99.00	45.90	91.96	47.60	92.30	49	2.6	56.8	JBBJZH000000000

Collectively, 87 C. *parakroppenstedtii*, 12 C. *pseudokroppenstedtii* and 2 other strains constituted the 101 CKC isolates.

### Detection of resistance genes based on WGS

**Figure 2:**
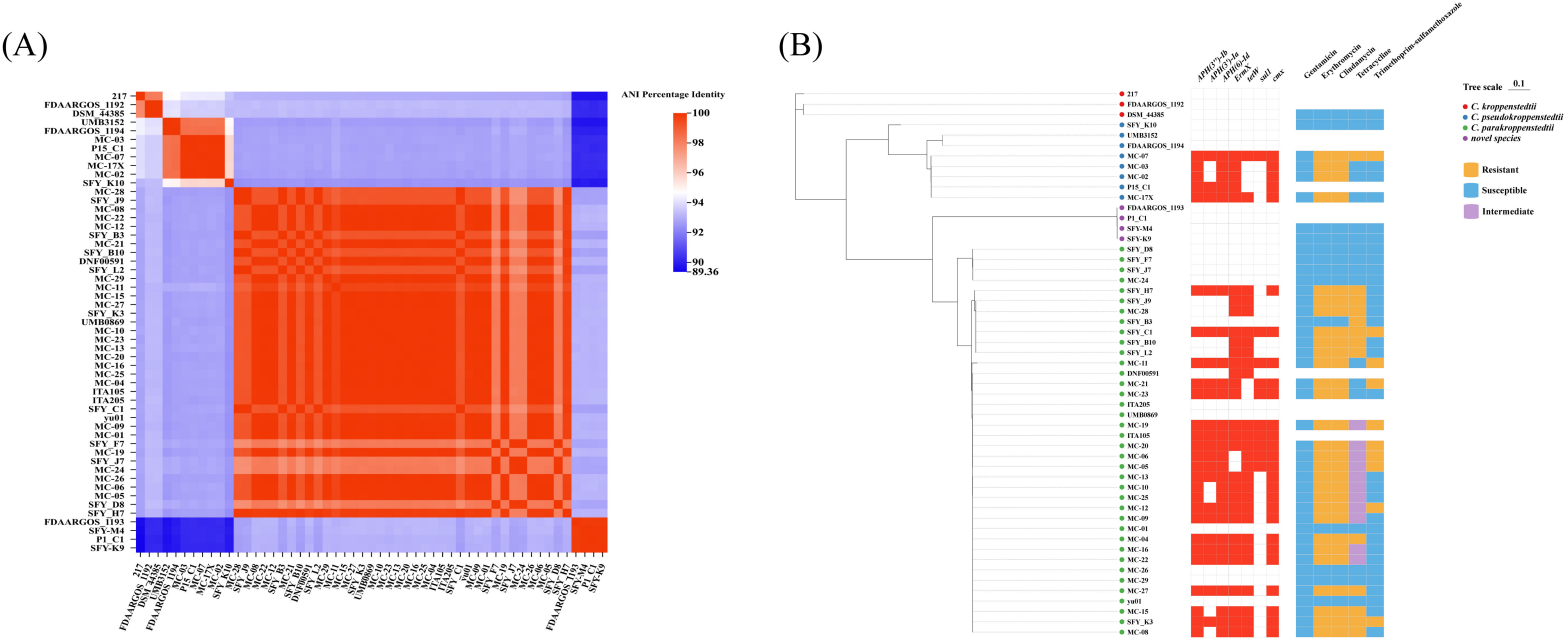
Phylogenetic analysis of 53 *Corynebacterium kroppenstedtii* Complex strains **(A)** SNP phylogenetic tree of CKC strains constructed based on maximum likelihood method and comparison of their drug resistance gene profiles; **(B)** Pairwise comparisons of ANIs of 53 CKCs and the three most closely type strains.

A total of 53 genome sequences of CKC strains were included in the resistance gene analysis, of which 40 genome sequences of CKC strains were obtained from the NCBI database. Pairwise comparisons of ANIs for 53 CKC strains and their three most closely type strains were shown on **Figure 2A**. As shown in **Figure 2B**, antibiotic resistance genes were compared among 53 isolates. Prediction of antimicrobial resistance genes has revealed distinct interspecies differences within the *Corynebacterium kroppenstedtii* complex. Antimicrobial resistance genes, namely *APH(3’)-Ia*, *APH(3’’)-Ib*, *APH(6)-Id*, *erm*(X), *tet*(W), sul1 and cmx were identified in most *C*. *parakroppenstedtii* and *C*. *pseudokroppenstedtii* isolates, while none were detected in *C*. *kroppenstedtii* or the novel species group (SFY-M4 and K9). However, within the *C*. *pseudokroppenstedtii* and *C*. p*aradokroppenstedtii* groups, no antibiotic resistance genes were predicted in isolates SFY-K10, UMB3152 and FDAARGOS_1194 and in SFY-D8, F7, J7, B3, MC-01,21,24,26, 29, UMB0869, ITA205 and yu01 respectively. Moreover, SFY-M4 and K9, which were classified as other CKC group from the ANI analysis above, shared identical resistance genes with P1_C1, FDAARGOS_1193.

### Phenotypic testing

All 101 isolates grew on Columbia blood agar were observed as smooth, circular, gray, convex and non-hemolytic colonies after 72h inoculation at 35°C, 5% CO_2_ atmosphere. Morphologically, these isolates were Gram-negative, rod-shaped, non-motile and non-spore-forming. The results of phenotypic testing are summarized in **Table 2**. All three groups of CKC isolates were lipophilic and catalase positive. On the contrary, no activity for nitrate reduction, urea hydrolysis or alkaline phosphatase. was detected among all isolates. Notably, 57% of the *C*. *parakroppenstedtii* group and 92% of the *C*. *pseudokroppenstedtii* group showed aesculin hydrolysis activity. However, this is negative for other CKC group (SFY-K9 and SFY-M4). For carbon source of acid production, all isolates were found to be able to produce acid from glucose but not from ribose or xylose. Only 66% of *C*. *parakroppenstedtii* group were able to produce acid from sucrose, whereas none of other CKC group (SFY-K9 and SFY-M4) use sucrose as carbon source just as *C*. *pseudokroppenstedtii* group.

**Table 2:** Phenotypic comparison of *C*. *parakroppenstedtii*, *C*. *pseudokroppenstedtii*, and related species isolates with their closest type strains.

Characteristics	Result for strains^a^					
	*C*. *parakroppenstedtii* (n = 87)	*C*. *pseudokroppenstedtii* (n = 12)	Other CKC stains (n = 2)	*C*. *kroppenstedtii* DSM 44385^T^	*C*. *parakroppenstedtii.* MC-26^T^	*C*. *pseudokroppenstedtii* MC-17X^T^
Lipophilism	100	100	100	+	+	+
Catalase	100	100	100	+	+	+
Nitrate reduction	0	0	0	–	–	–
Hydrolysis of aesculin	57	92	0	+	+	+
Hydrolysis of urea	0	0	0	_-	–	–
Enzyme activity						
Alkaline phosphatase	–	–	–	–	–	–
Acid production from						
Glucose	100	100	100	+	+	+
Sucrose	66	0	0	+	+	–
Ribose	0	0	0	–	–	–
Xylose	0	0	0	–	–	–

^a^ +, positive reaction; -, negative reaction.

### Antimicrobial susceptibility testing

Antimicrobial susceptibility testing was conducted on 101 isolates with confirmed genotypic identities from this study and 27 isolates from our previous study (Luo et al., 2022), following the standards set by the Clinical and Laboratory Standards Institute (CLSI). All of the isolates in *C*. *parakroppenstedtii* and *C*. *pseudokroppenstedtii* groups were sensitive to vancomycin, daptomycin, gentamicin and linezolid. Most *C*. *parakroppenstedtii* isolates were resistant to erythromycin (78.18%) and clindamycin (79.09%), while *C*. *pseudokroppenstedtii* isolated exhibited similarly high resistance rates (81.25% for the two antibiotics). Over 95% of the strains in the both groups showed intermediate susceptibility to penicillin. *C*. *parakroppenstedtii* group showed resistance to ceftriaxone in 20.00% and ciprofloxacin in 19.09%, while the resistance rates in *C. pseudokroppenstedii* group were 31.25% and 75.00%, respectively (**Table 3**). The Kirby–Bauer (KB) assay demonstrated that all *C*. *pseudokroppenstedtii* isolates (n = 12) and both isolates from the other CKC group (SFY-K9 and SFY-M4) were fully susceptible to rifampicin. In contrast, *C*. *parakroppenstedtii* isolates (n = 87) exhibited a high level of susceptibility to rifampicin, with 96.6% susceptible, 2.3% intermediate, and 1.1% resistant (**Supplementary Table S3**). In addition, another CKC group (SFY-K9 and M4) was sensitive to all antibiotics listed, with the exception of penicillin, which showed intermediate activity.

**Table 3:** Antimicrobial susceptibilities of *Corynebacterium parakroppenstedtii* and *Corynebacterium pseudokroppenstedtii*.

Antimicrobial agents	CLSI breakpoint(mg/L)^a^	*Corynebacterium parakroppenstedtii* MIC (mg/L) (n = 110)					*Corynebacterium pseudokroppenstedtii* MIC (mg/L) (n =16)				
		MIC_50_	MIC_90_	Susceptible %	Intermediate %	Resistant %	MIC_50_	MIC_90_	Susceptible %	Intermediate %	Resistant %
Penicillin	^a^S, ≤0.12; ^b^R, ≥4	0.5	2	3.64(4/110)	95.45(105/110)	0.01(1/110)	0.5	2	0.00(0/16)	100(16/16)	0.00(0/16)
Ceftriaxone	S, ≤1; R, ≥4	≤1	4	61.82(68/110)	18.18(20/110)	20.00(22/110)	2	4	18.75(3/16)	50(8/16)	31.25(5/16)
Cefepime	S, ≤1; R, ≥4	≤1	≤1	94.55(104/110)	44.55(5/110)	0.9(1/110)	≤1	≤1	100(16/16)	0.00(0/16)	0.00(0/16)
Meropenem	S, ≤0.25; R, ≥1	≤0.25	1	70.00(77/110)	26.36(29/110)	3.64(4/110)	≤0.25	≤0.25	100(16/16)	0.00(0/16)	0.00(0/16)
Vancomycin	S, ≤2	≤0.5	≤0.5	100(110/110)	0.00(0/110)	0.00(0/110)	≤0.5	≤0.5	100(16/16)	0.00(0/16)	0.00(0/16)
Daptomycin	S, ≤1	≤0.5	≤0.5	100(110/110)	0.00(0/110)	0.00(0/110)	≤0.5	≤0.5	100(16/16)	0.00(0/16)	0.00(0/16)
Gentamicin	S, ≤4; R, ≥16	≤4	≤4	99.10(109/110)	0.90(1/110)	0.00(0/110)	≤4	≤4	92.75(15/16)	6.25(1/16)	0.00(0/16)
Erythromycin	S, ≤0.5; R, ≥2	>8	>8	21.82(24/110)	0.00(0/110)	78.18(86/110)	>8	>8	18.75(3/16)	0.00(0/16)	81.25(13/16)
Ciprofloxacin	S, ≤1; R, ≥4	≤1	>4	80.00(88/110)	0.91(1/110)	19.09(21/110)	>4	>4	25.00(4/16)	0.00(0/16)	75.00(12/16)
Tetracycline	S, ≤4; R, ≥16	16	16	32.73(36/110)	10.91(12/110)	56.36(62/110)	16	16	50.00(8/16)	0.00(0/16)	50(8/16)
Clindamycin	S, ≤0.5; R, ≥4	>4	>4	20.91(23/110)	0.00(0/110)	79.09(87/100)	>4	>4	18.75(3/16)	0.00(0/16)	81.25(13/16)
Trimethoprim-sulfamethoxazole	S,≤2/38; R,>4/76	≤0.5/9.5	>4/76	74.55(82/110)	0.90(1/110)	24.55(27/110)	≤0.5/9.5	≤0.5/9.5	92.75(15/16)	0.00(0/16)	6.25(1/16)
Linezolid	S, ≤2	≤1	≤1	100(110/110)	0.00(0/110)	0.00(0/110)	≤1	≤1	100(16/16)	0.00(16/16)	0.00(0/16)
Ampicillin	–	0.5	>2	–	–	–	1	>2	–	–	–
Levofloxacin	–	≤2	>8	–	–		8	>8	–	–	–

^a^S, Susceptible; ^b^R, Resistant.

The “-” means no clinical breakpoint in CLSI M45.

### Clinical characteristics

Clinical characteristics of 101 mastitis patients in this study are provided in **Supplementary Table S1**, including treatment methods and prognosis results. Traditional Chinese medicine (TCM), antibiotics, and surgery were the most common treatments for mastitis. While most cases resulted in improvement or recovery, 4 worsen cases and 16 patients with recurrent mastitis were observed. These isolates were classified into the *C. parakroppenstedtii* (n = 87) and *C. pseudokroppenstedtii* (n = 12) groups as described above, and their clinical characteristics were compared by statistical analysis. As shown in **Table 4**, unilateral breast infections were more prevalent than bilateral infections in both two groups. Patients infected with *C*. *pseudokroppenstedtii* exhibited higher incidences of pus formation (66.7% vs. 44%) and greater recurrence rates (25% vs. 14.4%) compared to those infected with *C*. *parakroppenstedtii*. Conversely, *C*. *parakroppenstedtii* infections were more prevalent among parous women (95.8%) than *C*. *pseudokroppenstedtii* infections (87.5%). Clinical characteristics involving patient age, clinical presentation, wound diameter, lactation history, medical history, treatments, and ultrasound grading of two groups were displayed in **Table 4**. Isolates of other CKC group (SFY-K9 and SFY-M4) were not included due to a small sample size (n = 2). Additional details are provided in **Supplementary Table S1**.

**Table 4:** Demographics and clinical characteristics of patients infected by *Corynebacterium parakroppenstedtii.* and *Corynebacterium pseudokroppenstedtii*.

Characteristic	Total N = 99	*C. parakroppenstedtii* (n = 87)	*C.pseudokroppenstedtii* (n = 12)
Age in years, median (range)	32 (20-40)	32 (20-44)	33 (25-39)
Clinical presentation	n=99		
Nonlobular granulomatous mastitis	28	25 (28.7)	3 (25%)
Lobular granulomatous mastitis	71	62 (71.3)	9 (75%)
Laterality	n=99		
Left	51 (47.5)	43 (49.4%)	8 (66.8)
Right	39 (44.6)	37 (42.5%)	2 (16.6)
Bilateral	9 (9.9)	7 (8.1%)	2 (16.6)
Diameter ( (average, range) cm	6.8 (2-20)	6.9 (2-20)	5.8 (2-12.6)
Clinical manifestation			
Pain	83 (83.8)	73 (83.9)	10 (83.3)
Pus	48 (48.5)	40 (46.0)	8 (66.7)
Mass	94 (95.0)	83 (95.4)	11 (91.7)
Two or more of the above symptoms	89 (89.9)	78 (89.7)	11 (91.7)
Nipple retraction	15 (15.2)	14 (16.1)	1 (9.1%)
Nipple discharge	18 (18.2)	16 (18.4)	2 (18.2)
Maternity history	n=79		
Parous	75 (94.9)	68 (95.8%)	7 (87.5%)
Nulliparous	4 (5.1)	3 (4.2%)	1 (12.5%)
Lactation history	68 (68.9%)	62 (71.3%)	6 (50%)
Recurrence	99		
0	83 (83.8%)	74 (85.6%)	9 (75%)
≥1	16 (16.2%)	13 (14.4%)	3 (25%)
Medical History			
Hyperprolactinemia	10	8	2
Mammary gland tumor	5	4	1
Depression	8	6	2
Clinical Treatment with Medications	n=96		
Antibiotic	11 (11.5%)	9 (10.7%)	2 (16.7%)
Steroid	9 (9.4%)	8 (9.5%)	1 (8.3%)
TCM	5 (5.2%)	4 (4.8%)	1 (8.3%)
Two or more of the above Treatment	71 (73.9%)	63 (75%)	8 (66.7%)
Ultrasound grading	n=67		
1	2 (3.0%)	1 (1.8%)	1 (9.0%)
2	9 (13.4%)	6 (10.7%)	3 (27.3%)
3	26 (38.8%)	22 (39.3%)	4 (36.4%)
4	30 (44.8%)	27 (48.2%)	3 (27.3%)

## Discussion

In recent years, with the development of MALDI-TOF MS for clinical bacterial identification and the integration of molecular sequencing technologies into clinical practice, reports of mastitis associated with CKC infection have notably increased in China, particularly granulomatous mastitis (Zeng et al., 2022; Chen et al., 2023; Li et al., 2023; Liang et al., 2024; Xiao and Zhao, 2024; Zeng et al., 2024). This condition significantly compromises the quality of life of patients due to its prolonged course of illness (Pala et al., 2022) and high recurrence rate (15.4 -24.8%) (Azizi et al., 2020). Given the recent emergence of *C*. *parakroppenstedtii* and *C*. *pseudokroppenstedtii*, a comprehensive analysis comparing their clinical, epidemiology and microbiological characteristics is needed to enhance clinicians’ awareness of differential diagnoses. In this study, we analyzed 101 clinical breast specimens collected from 2021 to 2023 in China representing the largest and most epidemiological investigation of *C*. *parakroppenstedtii* and *C*. *pseudokroppenstedtii* infection. Comprehensive molecular analysis revealed that *C*. *parakroppenstedtii* (86%) is the primary pathogen responsible for CKC-related mastitis, followed by *C*. *pseudokroppenstedtii* (12%), while no *C*. *kroppenstedtii* was detected—challenging the traditional view of its primary role. Notably, GM was the most common presentation for both novel species, with parous women exhibiting significantly higher susceptibility, suggesting obstetric history as a potential key risk factor. Clinical symptoms, including pain, pus formation, and palpable masses, were consistent with those reported in cases of mastitis caused by *C*. *kroppenstedtii* (Zeng et al., 2022). underscoring the need for species-level differentiation in diagnosis.

Despite increasing studies on mastitis, comparative analyses among different species often overlook CKC due to their presence of analogous morphological and genomic characteristics. Our study is the first to delineate distinct clinical profiles for *C*. *parakroppenstedtii* and *C*. *pseudokroppenstedtii*. Patients infected with *C*. *pseudokroppenstedtii* are more prone to develop abscesses and experience relapse compared to those infected with *C*. *parakroppenstedtii*, indicating greater clinical severity. Additionally, *C*. *parakroppenstedtii* infections was more prevalent in parous women and strongly associated with GM. These findings underscore the need for clinicians to realize that mastitis caused by different species of *Corynebacterium* may present distinct epidemiological characteristics, which are crucial for clinical diagnosis and treatment.

While both *C*. *parakroppenstedtii* and *C*. *pseudokroppenstedtii* isolates exhibited properties generally consistent with their respective type strains, we observed variability in sucrose utilization among *C*. *parakroppenstedtii* strains, consistent with findings from previous studies (Luo et al., 2022; Xiao and Zhao, 2024). Additionally, two unidentified isolates, classified as a distinct CKC group, exhibited distinct phenotypes compared to the *C. parakroppenstedtii* and *C. pseudokroppenstedtii* groups, as they were unable to hydrolyze aesculin or produce acid from sucrose. However, a larger sample size is needed to confirm whether these isolates represent a distinct CKC phenotype.

In clinical practice, accurately identifying bacterial strains is only one step toward effective patient management; antibiotic susceptibility testing is equally critical for guiding clinicians in selecting targeted therapy. Studies have shown that detailed antimicrobial profiling improves treatment outcomes and minimizes the emergence of resistant strains, which can be particularly important in managing infections caused by lipophilic *Corynebacterium* species (Dobinson et al., 2015; Zeng et al., 2022; Li et al., 2023; Zeng et al., 2024). As observed in the clinical cases examined in the present study, the empirical treatments that doctors prescribe prior to the availability of antibiotic susceptibility results include broad-spectrum antibiotics, e.g. cefuroxime and levofloxacin. The literature has also demonstrated the efficacy of cefuroxime and levofloxacin in the treatment of infectious mastitis (Zeng et al., 2022). However, growing evidence suggests that lipophilic antibiotics (e.g., rifampicin, doxycycline, and clarithromycin) may offer therapeutic advantages because of their enhanced ability to penetrate lipid-rich granulomata effectively (Paviour et al., 2002; Qiu et al., 2021; Zeng et al., 2022). Additionally, the use of rifampicin has also been demonstrated to reduce the recurrence rate of mastitis (Qiu et al., 2021; Li et al., 2023). Moreover, the clinical cases have been demonstrated that the early accurate recognition and timely initiation of appropriate antibiotic therapy are effective and necessary for the inhibition of *Corynebacterium* (Yu et al., 2016; Wolfrum et al., 2018; Chen et al., 2023). As the abundance of *Corynebacterium* decreases, clinical outcomes can be improved, including a reduced incidence of abscess formation and alleviation of symptoms (Johnstone et al., 2017; Zeng et al., 2022; Chen et al., 2023) However, data on antibiotics for the newly identified species, *C*. *parakroppenstedtii* and *C*. *pseudokroppenstedtii*, was limited and it has not been reported whether there are differences in antimicrobial susceptibility characteristics between these novel species.

Comparing the resistance profiles of *C*. *parakroppenstedtii* and *C*. *pseudokroppenstedtii* can offer valuable insights into developing more effective therapeutic strategies for managing infections. To explore the differences in antibiotic susceptibility between the two species, we performed antibiotic susceptibility testing alongside an analysis of antibiotic resistance genes. First, in an antimicrobial susceptibility testing, the isolates of CPK in this study exhibited high susceptibility to vancomycin, daptomycin, gentamicin, linezolid, cefepime, meropenem and ceftriaxone, intermediate activity to penicillin, partial susceptibility to ciprofloxacin, tetracycline and trimethoprim-sulfamethoxazole, and mostly resistance to erythromycin and clindamycin. These findings are generally consistent with those of previous research, with some exceptions where isolates showed high resistance to ciprofloxacin and penicillin (Luo et al., 2022; Xiao and Zhao, 2024; Zeng et al., 2024). Variations of antimicrobial susceptibility across to studies may be attributed differences in sample sizes (Xiao and Zhao, 2024; Zeng et al., 2024). The majority of strains in *C. pseudokroppenstedtii* group display comparable profiles to those observed in the limited existing literature (Luo et al., 2022; Xiao and Zhao, 2024). Notably, in this study, it seems that *C*. *pseudokroppenstedtii* exhibits higher levels of resistance to ceftriaxone and ciprofloxacin compared to *C. parakroppenstedtii*, underscoring the importance of accurate species-level identification for CKC strains and highlight the necessity of evidence-based antibiotic selection. Nevertheless, additional large-scale research is necessary to validate these findings and further refine treatment strategies.

Then, the antimicrobial resistance genes were compared on selected isolates subjected to WGS. The resistance genes including the macrolide and lincosamide resistance gene *erm*(X), the tetracycline resistance gene *tet*(W), sulfonamide resistance gene *sul1*, the aminoglycoside-modifying enzyme coding genes *APH(3’)-Ia*, *APH(3’’)-Ib* and *APH(6)-Id*, the efflux-pump encoding chloramphenicol-resistance gene *cmx* were predicted from draft genome sequence of CKCs, most of which have been previously reported in other literatures (Fernández-Natal et al., 2016; Luo et al., 2022). We observed a correlation between antibiotic resistance genes and their clustering within branches of the SNP tree. By comparing these genomic findings with the results of the susceptibility tests performed earlier, we identified several notable findings: (i) Most of isolates from *C*. *parakroppenstedtii* and *C*. *pseudokroppenstedtii* groups harbored *erm(X)*, which potentially explains why these isolates showed resistance to erythromycin and clindamycin. (ii) The resistance gene sul1 is likely to be responsible to resistance to sulfonamide, based on the high consistence in phenotype and genotype of isolates in *C*. *parakroppenstedtii* and *C*. *pseudokroppenstedtii* groups. (iii) Isolates harboring *tet*(W) were observed intermediate or resistant to tetracycline, which also interprets the antimicrobial resistance mechanism of CKC in this study.

Furthermore, other antimicrobial phenotypes could be explained as followings: (i) No quinolone resistance gene were identified based on the draft genome sequences with 90% identity and coverage in this study. Notably, it has been reported in other *Corynebacterium* bacteria that the quinolone-resistant *Corynebacterium* were due to *gyrA* mutations (Wang et al., 2021). Therefore, it would be reasonable for further investigation to perform polymerase chain reaction (PCR), sequencing and check point mutations on *gyrA*. (ii) The *APH(3’)-Ia*, *APH(3’’)-Ib* and *APH(6)-Id* genes were detected in over 50% of isolates, yet they remained susceptibility to gentamicin. The resistance to aminoglycosides may be also due to other resistance genes such as the *aac(3)-XI*, a gene encoding a new aminoglycoside 3-N acetyl transferase (Navas et al., 2016), which have been reported in other *Corynebacterium* species. (iii) Most of the isolates showed intermediate (equivalent to MIC90) to penicillin, and partial isolates conferred resistance to ceftriaxone. Only very few C. *parakroppenstedtii* show resistance to cefepime or meropenem, implicating very few C. *parakroppenstedtii* resistance to 4^th^ generation cephalosporin or carbapenem antimicrobials. (iv) Furthermore, few drug resistance genes were detected in another CKC group (SFY-M4 and SFY-K9), indicating their susceptibility to the antibiotic drugs. These findings provide attention for epidemiology, microbiological characteristics and antimicrobial resistant mechanisms of CKC isolate, highlighting the importance of rational treatments of antibiotics for mastitis cases.

Collectively, these findings suggest that the prediction of drug resistance genes can, facilitate clinical comprehension of the drug resistance characteristics of strains. However, the presence of drug resistance genes does not necessarily correlate with the manifestation of phenotypic resistance. Instead, this discrepancy may be attributed to variations in gene expression levels among isolates or the incomplete nature of the draft genome sequences, as mentioned in Luo Q et al (Luo et al., 2022).

Based on previous literature reports, there is currently no unified consensus or standard for the treatment of mastitis associated with *Corynebacterium* infections. In this study, our patients received various treatment strategies based on individual clinical assessments, including antibiotics, steroid, TCM, and surgery. These methods were applied either as standalone treatments or in various combinations. Most patients received combination therapy and achieved good therapeutic outcomes. Among these cases, approximately 70% of patients received antibiotic treatments, such as rifampicin, levofloxacin, and cefuroxime, most of which effectively suppressed the progression of inflammation. Additionally, nearly one-third of patients were treated with TCM, with no cases of disease worsening observed. These results underscore that a personalized, multimodal treatment approach represents a feasible and effective strategy for the clinical management of *Corynebacterium* CKC–associated mastitis.

## Conclusions

In conclusion, this study, encompassing the largest sample size to date, provides critical insights into the distinct genomic attributes, antimicrobial susceptibilities, and clinical epidemiology of two CKC species isolated from our clinical breast specimens. Our findings enhance the current- understanding of the CKC and underscore the importance of vigilant isolation and accurate identification of *Corynebacterium* species in breast samples. Furthermore, reliable taxonomic characterization, along with comparative assessments of clinical features and antimicrobial susceptibilities of these potential pathogens, will be crucial for informing effective diagnostic and therapeutic strategies for mastitis.

## Data Availability

The datasets presented in this study can be found in online repositories. The names of the repository/repositories and accession number(s) can be found in the article/**Supplementary Material**.
