# Supplementary material for: Emerging mastitis-associated Corynebacterium parakroppenstedtii and Corynebacterium pseudokroppenstedtii: clinical, microbiological, and epidemiological features from a two-year study in Guangdong, China
Source: Front Cell Infect Microbiol. 2026 Jan 14;15:1723551. doi: 10.3389/fcimb.2025.1723551 (PMC12847403; doi:10.3389/fcimb.2025.1723551)
Supplement: Supplementary file 1 [file Table1.doc]

**Online supplementary files for**

**Emerging mastitis-associated *Corynebacterium* *parakroppenstedtii* and *C*. *pseudokroppenstedtii*: clinical, microbiological, and epidemiological features from a two-year study in Guangdong, China**

**Authors**

**Minling Zheng,^1#^ Qiongdan Mai,^1#^ Yasha Luo,^1#^ Xiaowei Chen,^2^ Weiming Lai,^1^ Junfei Guo,^1^ Yanting Qin,^1,3^ Lingling Tang,^1^ Zhiyu Li,^1,3^ Hongyu Li,^1^ Wenyu Deng,^1^ Pinghua Qu,^4*^ Mingyong Luo^1*^**

^#^ Minling Zheng, Qiongdan Mai and Yasha Luo contributed equally to this article

**Authors affiliations**

^1^Department of Clinical Laboratory, Guangdong Women and Children Hospital,

Guangzhou, China

^2^Department of Laboratory Medicine, Panyu Hospital of Chinese Medicine, Guangzhou, China

^3^ Guangzhou Medical University, Guangzhou, China.

^4^School of Medicine, Foshan University, Foshan, China

**Corresponding authors**

Dr. Mingyong Luo

Email: luo-my@163.com

Dr. Pinghua Qu

Email: ququtdr@163.com

**Table S1** **Clinical characteristics of mastitis patients infected with *C*. *parakroppenstedtii*, *C*. *pseudokroppenstedtii* and related species in Guangdong, China.**

| Strain no. | Age (yr)/sex^a^ | Specimen source | Diagnosis^b^ | Comorbidity | Treatment^c^ | Prognosis |
| --- | --- | --- | --- | --- | --- | --- |
| *C*.*pseudokroppenstedtii* group |  |  |  |  |  |  |
| SFY-A9 | 34/F | Pus | GM | No record. | TCM ；Antibiotic (rifampicin) | Improvement |
| SFY-A10 | 33/F | Pus | GM | endemic goiter | Surgery，rifampicin, methylprednisolone; Red Gold Nodule-Reducing Capsules | Improvement, but Recurrence after 2 months |
| SFY-B5 | 39/F | Pus | GM | Hyperprolactinemia | Surgery，antibiotic(levofloxacin， rifampicin ,Azithromycin); methylprednisolone ；bromocriptine；Red Gold Nodule-Reducing Capsules | Improvement, but Recurrence after 6 months |
| SFY-B8 | 26/F | Pus | M | Atypical depression | Antibiotic (rifampicin, ethambutol, isoniazide); Red Gold Nodule-Reducing Capsules | Improvement |
| SFY-E8 | 31/F | Pus | M | No record. | Antibiotic (rifampicin, ethambutol, isoniazide); Red Gold Nodule-Reducing Capsules | Recovery |
| SFY-H2 | 37/F | Pus | GM | Fibroadenoma of breast；Alpha thalassemia | Methylprednisolone；Red Gold Nodule-Reducing Capsules | Improvement |
| SFY-I5 | 34/F | Pus | GM | Hashimoto's thyroiditis | Methylprednisolone; antibiotic (levofloxacin, Azithromycin); Bromocriptine | Recurrence |
| SFY-J1 | 32/F | Pus | GM | Schizophrenia | TCM | Improvement |
| SFY-K10 | 25/F | Pus | M | Hyperprolactinemia；depression | Antibiotic (levofloxacin); TCM | Improvement |
| SFY-L9 | 33/F | Pus | GM | Unknown | Antibiotic (levofloxacin )；TCM；prednisone | Recurrence |
| SFY-N3 | 39/F | Pus | GM | diabetes | Antibiotic (Azithromycin; levofloxacin)  Surgery  Red Gold Nodule-Reducing Capsules, | Improvement |
| SFY-01 | 29/F | Pus | GM | Fatty liver | Surgery；Antibiotic (levofloxacin )；Methylprednisolone | Improvement |
| *C*. *parakroppenstedtii* group |  |  |  |  |  |  |
| SFY-B3 | 27/F | Pus | M | Erythema nodosum；urticaria；Pregnancy | Surgery； Antibiotic (cefuroxime axetil)；Methylprednisolone | Recovery |
| SFY-B10 | 32/F | Pus | PCM | No record. | TCM ；Antibiotic (levofloxacin) | Improvement |
| SFY-C1 | 44/F | Pus | GM | Hyperprolactinemia | Antibiotic(rifampicin, ethambutol, isoniazide)；  Methylprednisolone；Bromocriptine | Improvement |
| SFY-H7 | 36/F | Pus | M | No record. | Antibiotic(rifampicin, cefuroxime axetil)，TCM，punctures | Recurrence |
| SFY-J9 | 28/F | Pus | M | No record. | Antibiotic (levofloxacin, cefuroxime axetil), TCM; Red Gold Nodule-Reducing Capsules | Recurrence |
| SFY-K3 | 31/F | Pus | GM | Hyperprolactinemia | Antibiotic (rifampicin, ethambutol, isoniazide); Bromocriptine; Red Gold Nodule-Reducing Capsules | Recurrence |
| SFY-L2 | 35/F | Pus | GM | No record. | Antibiotic(levofloxacin, ethambutol, isoniazide)；TCM | Improvement |
| SFY-A5 | 34/F | Pus | M | Hyperprolactinemia;  Benign tumor of breast | Antibiotic(levofloxacin); methylprednisolone; bromocriptine；  Red Gold Nodule-Reducing Capsules | Improvement |
| SFY-A6 | 34/F | Pus | GM | Schizophrenia; HBV | TCM; Methylprednisolone | Recovery |
| SFY-A7 | 32/F | Pus | GM | Benign tumor of breast | Surgery; methylprednisolone; Antibiotic (cefuroxime axetil);  Red Gold Nodule-Reducing Capsules | Recovery |
| SFY-A8 | 34/F | Pus | GM | No record. | Methylprednisolone; Antibiotic (rifampicin, levofloxacin) | Improvement |
| SFY-B1 | 32/F | Pus | GM | No record. | Methylprednisolone; Antibiotic (rifampicin); bromocriptine | Recovery |
| SFY-B2 | 32/F | Pus | GM | No record. | Antibiotic (cefuroxime axetil) | Improvement |
| SFY-B6 | 31/F | Pus | GM | Hyperprolactinemia; | Surgery，antibiotic(rifampicin, ethambutol, isoniazide)； methylprednisolone ；bromocriptine； | Worsen |
| SFY-B9 | 36/F | Pus | M | Unknown | Antibiotic（levofloxacin） | Worsen |
| SFY-C2 | 34/F | Pus | GM | Hyperuricemia | Surgery，antibiotic(levofloxacin)； methylprednisolone | Recovery |
| SFY-C3 | 33/F | Pus | GM | No record. | Surgery，antibiotic(levofloxacin)； methylprednisolone | Improvement |
| SFY-C5 | 38/F | Pus | GM | No record. | antibiotic(rifampicin, ethambutol, isoniazide)；TCM; methylprednisolone | Unknown |
| SFY-C6 | 38/F | Pus | GM | No record. | antibiotic(levofloxacin)；methylprednisolone | Improvement |
| SFY-C7 | 40/F | Pus | GM | depression | TCM | Improvement |
| SFY-C8 | 31/F | Pus | M | acute vaginitis | Antibiotic (rifampicin; isoniazide) | Improvement |
| SFY-C10 | 34/F | Pus | GM | No record. | TCM; Antibiotic (rifampicin; levofloxacin) | Improvement |
| SFY-D5 | 31/F | Pus | GM | Hyperprolactinemia;  Benign pituitary tumor | TCM, methylprednisolone, bromocriptine；Predisone; Red Gold Nodule-Reducing Capsules | Improvement |
| SFY-D6 | 37/F | Pus | GM | No record. | Methylprednisolone, bromocriptine; Red Gold Nodule-Reducing Capsules | Improvement |
| SFY-D8 | 27/F | Pus | GM | No record. | Methylprednisolone; Antibiotic (levofloxacin); bromocriptine | Improvement |
| SFY-D10 | 40/F | Pus | M | Hyperprolactinemia; depression | TCM, Antibiotic (rifampicin; ethambutol) | Improvement |
| SFY-E2 | 36/F | Pus | GM | No record. | Predisone | Improvement |
| SFY-E3 | 34/F | Pus | GM | No record. | Antibiotic (rifampicin, ethambutol, isoniazide) | Recurrence |
| SFY-E5 | 32/F | Pus | GM | HBV carrier | Methylprednisolone | Recurrence |
| SFY-E6 | 27/F | Pus | GM | No record. | Methylprednisolone;  Antibiotic (rifampicin, ethambutol, isoniazide) | Recovery |
| SFY-E7 | 25/F | Puncture fluid | GM | fibroadenoma | Surgery; antibiotic(levofloxacin);  Methylprednisolone | Improvement |
| SFY-E10 | 25/F | Pus | GM | No record. | TCM, antibiotic(levofloxacin) | Improvement |
| SFY-F2 | 30/F | Pus | GM | No record. | Antibiotic(levofloxacin);  Red Gold Nodule-Reducing Capsules | Recurrence |
| SFY-F4 | 31/F | Pus | GM | No record. | Methylprednisolone; Antibiotic(rifampicin); Red Gold Nodule-Reducing Capsules | Recovery |
| SFY-F5 | 31/F | Pus | GM | No record. | Methylprednisolone; Antibiotic(levofloxacin); TCM | Recovery |
| SFY-F6 | 26/F | Pus | M | fibroadenoma | Predisone; Antibiotic(rifampicin);  Surgery | Improvement |
| SFY-F7 | 31/F | Pus | GM | No record. | Methylprednisolone; Antibiotic(levofloxacin);  Red Gold Nodule-Reducing Capsules | Unknown |
| SFY-F8 | 34/F | Pus | M | No record. | Methylprednisolone; Red Gold Nodule-Reducing Capsules | Improvement |
| SFY-F9 | 30/F | Pus | GM | No record. | Methylprednisolone | Improvement |
| SFY-F10 | 43/F | Pus | M | No record. | Methylprednisolone; TCM | Improvement |
| SFY-G1 | 31/F | Pus | M | No record. | Methylprednisolone; TCM | Unknown |
| SFY-G2 | 25/F | Pus | GM | No record. | Prednisone | Recurrence |
| SFY-G3 | 35/F | Pus | GM | polycystic ovary syndrome | Methylprednisolone; Antibiotic(levofloxacin);  Surgery | Recovery |
| SFY-G5 | 27/F | Pus | GM | No record. | Prednisone; Red Gold Nodule-Reducing Capsules | Improvement |
| SFY-G6 | 31/F | Pus | GM | No record. | Methylprednisolone; Surgery; Antibiotic (rifampicin, ethambutol, isoniazide) | Recovery |
| SFY-G8 | 27/F | Puncture fluid | GM | No record | Methylprednisolone; Surgery | Improvement |
| SFY-G9 | 38/F | Pus | GM | No record. | Antibiotic (rifampicin, ethambutol, isoniazide; Cephalosporin); punctures | Improvement |
| SFY-G10 | 36/F | Pus | GM | Pregnancy | No medication used | Worsen |
| SFY-H3 | 37/F | Pus | M | Hyperprolactinemia;  thyroid nodule | Methylprednisolone; Antibiotic (rifampicin, isoniazide,); TCM; bromocriptine | Improvement |
| SFY-H4 | 30/F | Pus | M | No record. | Methylprednisolone; Antibiotic(levofloxacin);  bromocriptine | Improvement |
| SFY-H5 | 29/F | Pus | GM | No record. | Methylprednisolone; Antibiotic (rifampicin); TCM | Improvement |
| SFY-H6 | 30/F | Pus | GM | No record. | Surgery; Methylprednisolone;  Antibiotic (levofloxacin, rifampicin) | Improvement |
| SFY-H8 | 40/F | Pus | M | No record. | Methylprednisolone; TCM | Recurrence |
| SFY-H9 | 20/F | Pus | M | Pregnancy | Methylprednisolone; bromocriptine | Improvement |
| SFY-H10 | 27/F | Pus | GM | Hyperuricemia;  Hyperbilirubinemia  hyponatremia | Methylprednisolone; Surgery; | Improvement |
| SFY-I1 | 30/F | Pus | GM | No record. | Methylprednisolone; Surgery; Antibiotic(levofloxacin); | Recurrence |
| SFY-I2 | 33/F | Pus | M | Pregnancy | No medication used | Unknown |
| SFY-I6 | 29/F | Pus | GM | No record | Surgery；prednisone | Improvement |
| SFY-I7 | 32/F | Pus | GM | No record | Methylprednisolone; TCM；  Antibiotic (rifampicin, ethambutol, isoniazide) | Improvement |
| SFY-I8 | 22/F | Pus | Plasma cell mastitis | No record | Antibiotic (Cefuroxime axetil) | Improvement |
| SFY-J2 | 32/F | Pus | GM | HBV carrier | Methylprednisolone; Antibiotic (azithromycin);  TCM; Red Gold Nodule-Reducing Capsules | Improvement |
| SFY-J3 | 35/F | Pus | M | No record | Methylprednisolone; Antibiotic (azithromycin);  TCM; Red Gold Nodule-Reducing Capsules | Improvement |
| SFY-J4 | 44/F | Pus | GM | Heart disease | TCM; Antibiotic (levofloxacin, rifampicin) | Improvement |
| SFY-J5 | 36/F | Pus | GM | Cervicitis | Surgery; Methylprednisolone; Antibiotic (levofloxacin) | Improvement |
| SFY-J7 | 30/F | Pus | GM | depression | TCM | Recovery |
| SFY-J10 | 35/F | Pus | GM | Hyperthyroidism | Methylprednisolone；TCM | Improvement |
| SFY-K1 | 30/F | Pus | M | Schizophrenia | Surgery；  Antibiotic (levofloxacin，azithromycin) | Improvement |
| SFY-K4 | 38/F | Pus | GM | Hyperprolactinemia;  Benign pituitary tumor | Antibiotic (levofloxacin，azithromycin);  TCM; bromocriptine | Improvement |
| SFY-K5 | 42/F | Pus | GM | No record | Predisone; Red Gold Nodule-Reducing Capsules | Recurrence |
| SFY-K6 | 37/F | Pus | GM | tuberculosis | Methylprednisolone; Surgery; TCM;  Antibiotic (levofloxacin); | Improvement |
| SFY-K7 | 32/F | Pus | GM | No record | Methylprednisolone; TCM | Improvement |
| SFY-K8 | 34/F | Pus | GM | No record | Antibiotic (rifampicin, isoniazide) | Recurrence |
| SFY-L1 | 26/F | Pus | M | No record | No medication used | Unknown |
| SFY-L3 | 28/F | Pus | GM | No record | Antibiotic (rifampicin, ethambutol, isoniazide, levofloxacin); Methylprednisolone | Improvement |
| SFY-L6 | 34/F | Pus | GM | Pregnancy; Hypothyroidism | Cefuroxime; clindamycin | Worsen |
| SFY-M1 | 35/F | Pus | M | No record | Antibiotic (Cefuroxime) | Improvement |
| SFY-M2 | 36/F | Pus | M | No record | Methylprednisolone; TCM;  Antibiotic (Cefuroxime) | Improvement |
| SFY-M3 | 43/F | Pus | GM | Hyperprolactinemia; depression;diabetes | TCM; Antibiotic (Cefuroxime, levofloxacin) | Recovery |
| SFY-M6 | 36/F | Pus | GM | No record | TCM | Recovery |
| SFY-M9 | 26/F | Pus | M | No record | Methylprednisolone； Antibiotic (azithromycin levofloxacin)；Red Gold Nodule-Reducing Capsules | Improvement |
| SFY-M10 | 26/F | Pus | GM | No record | Methylprednisolone；Antibiotic (rifampicin；levofloxacin)；Surgery | Improvement |
| SFY-N1 | 30/F | Pus | GM | Hyperprolactinemia;  Benign pituitary tumor | TCM | Recovery |
| SFY-N5 | 42/F | Pus | GM | No record | Methylprednisolone;  Antibiotic (rifampicin, isoniazide) | Recovery |
| SFY-N6 | 32/F | Pus | GM | No record | Surgery；Methylprednisolone; Red Gold Nodule-Reducing Capsules | Improvement |
| SFY-N9 | 32/F | Pus | GM | No record | Methylprednisolone; Antibiotic (rifampicin, levofloxacin),；TCM；Red Gold Nodule-Reducing Capsules | Improvement |
| SFY-02 | 36/F | Pus | GM | diabetes | Methylprednisolone; Antibiotic (azithromycin；levofloxacin)；Red Gold Nodule-Reducing Capsules | Recovery |
| SFY-04 | 35/F | Pus | M |  | Methylprednisolone；Antibiotic(levofloxacin)；Red Gold Nodule-Reducing Capsules | Recurrence |
| Other CKC group |  |  |  |  |  |  |
| SFY-K9 | 40/F | Pus | GM | No record | Methylprednisolone；Red Gold Nodule-Reducing Capsules | Unknown |
| SFY-M4 | 35/F | Pus | GM | No record | Methylprednisolone，Antibiotic (rifampicin, ethambutol, isoniazide, Vancomycin); Surgery | Improvement |

^a^F, female.

^b^GLM, granulomatous lobular mastitis; PCM, plasma cell mastitis; M, mastitis.

^c^Surgery included abscess incision drainage and debridement. TCM, traditional Chinese medicine.

**Table S2 Polyphasic characterization of *C*. *parakroppenstedtii*, *C*. *pseudokroppenstedtii*, and related species isolates compared to their closest type strains.**

|  | Strain identified by MALDTOF-MS in database |  |  | Partial 16S rRNA gene Similarity (%)^a^ | | |  | Partial rpoB gene Similarity (%)^a^ | | |  | Partial fusA gene Similarity (%)^a^ | | |
| --- | --- | --- | --- | --- | --- | --- | --- | --- | --- | --- | --- | --- | --- | --- |
| Strain | Biotyper database | score value |  | MC-17X^T^ | MC-26^T^ | DSM44385^T^ |  | MC-17X^T^ | MC-26^T^ | DSM44385^T^ |  | MC-17X^T^ | MC-26^T^ | DSM44385^T^ |
| ***C*. *pseudokroppenstedtii* group** | |  |  |  |  |  |  |  |  |  |  |  |  |  |
| SFY-A9 | Ckr CCUG 44504 | 2.144 |  | 99.93 | 99.48 | 99.64 |  | 100.00 | 96.12 | 95.30 |  | 100.00 | 97.57 | 97.15 |
| SFY-A10 | Ckr CCUG 44504 | 2.138 |  | 100.00 | 99.56 | 99.71 |  | 100.00 | 96.12 | 95.30 |  | 99.89 | 97.47 | 96.90 |
| SFY-B5 | Ckr CCUG 44504 | 2.149 |  | 100.00 | 99.56 | 99.57 |  | 100.00 | 96.13 | 95.79 |  | 99.90 | 97.51 | 97.13 |
| SFY-B8 | Ckr CCUG 44504 | 1.696 |  | 100.00 | 99.56 | 99.78 |  | 100.00 | 96.12 | 95.53 |  | 99.59 | 97.16 | 96.52 |
| SFY-E8 | Ckr CCUG 44504 | 2.309 |  | 100.00 | 99.56 | 99.71 |  | 100.00 | 96.12 | 95.53 |  | 99.29 | 96.86 | 96.25 |
| SFY-H2 | Ckr CCUG 44504 | 2.215 |  | 100.00 | 99.56 | 99.79 |  | 100.00 | 96.58 | 96.21 |  | 99.90 | 97.44 | 97.14 |
| SFY-I5 | Ckr CCUG 44504 | 2.346 |  | 100.00 | 99.56 | 99.79 |  | 100.00 | 96.34 | 95.96 |  | 99.90 | 97.49 | 97.10 |
| SFY-J1 | Ckr CCUG 44504 | 2.087 |  | 100.00 | 99.56 | 99.78 |  | 99.42 | 97.10 | 96.12 |  | 99.80 | 97.37 | 96.94 |
| SFY-K10 | Ckr DSM 44385T | 1.223 |  | 99.93 | 99.56 | 99.71 |  | 97.89 | 96.31 | 96.46 |  | 96.05 | 95.95 | 95.50 |
| SFY-L9 | Ckr CCUG 44504 | 1.875 |  | 100.00 | 99.56 | 99.79 |  | 100.00 | 96.55 | 95.70 |  | 99.29 | 96.86 | 96.51 |
| SFY-N3 | Ckr CCUG 44504 | 2.203 |  | 100.00 | 99.56 | 99.78 |  | 99.74 | 96.33 | 96.15 |  | 99.90 | 97.46 | 97.00 |
| SFY-01 | Ckr CCUG 44504 | 2.199 |  | 99.93 | 99.48 | 99.64 |  | 100.00 | 96.57 | 96.61 |  | 99.09 | 96.66 | 96.27 |
| ***C*. *parakroppenstedtii* group** | |  |  |  |  |  |  |  |  |  |  |  |  |  |
| SFY-A5 | Ckr CCUG 44504 | 1.541 |  | 99.56 | 100.00 | 99.57 |  | 96.08 | 99.74 | 95.75 |  | 97.50 | 99.90 | 99.57 |
| SFY-A6 | Ckr CCUG 44504 | 1.595 |  | 99.56 | 100.00 | 99.50 |  | 96.13 | 100.00 | 96.04 |  | 97.24 | 99.69 | 99.50 |
| SFY-A7 | Ckr CCUG 44504 | 1.553 |  | 99.56 | 100.00 | 99.57 |  | 96.13 | 100.00 | 95.80 |  | 97.61 | 100.00 | 99.57 |
| SFY-A8 | Ckr CCUG 44504 | 1.75 |  | 99.93 | 99.48 | 99.64 |  | 96.12 | 100.00 | 95.78 |  | 97.44 | 99.90 | 99.64 |
| SFY-B1 | Ckr CCUG 61180 | 1.713 |  | 99.56 | 100.00 | 99.57 |  | 96.12 | 99.49 | 95.78 |  | 97.37 | 99.80 | 99.57 |
| SFY-B2 | Ckr CCUG 49276 | 1.532 |  | 99.56 | 100.00 | 99.56 |  | 96.12 | 100.00 | 95.78 |  | 97.25 | 99.69 | 99.56 |
| SFY-B3 | Ckr CCUG 44504 | 1.817 |  | 99.56 | 100.00 | 99.57 |  | 96.38 | 97.16 | 97.71 |  | 97.46 | 97.36 | 99.57 |
| SFY-B6 | Ckr CCUG 44504 | 1.998 |  | 99.56 | 100.00 | 99.36 |  | 96.12 | 100.00 | 96.03 |  | 97.06 | 99.49 | 99.36 |
| SFY-B9 | Ckr CCUG 44504 | 1.767 |  | 99.56 | 100.00 | 99.57 |  | 96.13 | 100.00 | 95.79 |  | 97.17 | 99.60 | 99.57 |
| SFY-B10 | Ckr CCUG 61180 | 1.638 |  | 99.56 | 100.00 | 99.57 |  | 97.16 | 96.38 | 97.03 |  | 97.47 | 97.37 | 99.57 |
| SFY-C1 | Ckr CCUG 61180 | 1.567 |  | 99.56 | 100.00 | 99.50 |  | 97.16 | 96.38 | 97.03 |  | 97.57 | 97.47 | 99.50 |
| SFY-C2 | Ckr CCUG 61180 | 1.943 |  | 99.56 | 100.00 | 99.57 |  | 96.13 | 100.00 | 95.79 |  | 97.06 | 99.49 | 99.57 |
| SFY-C3 | Ckr CCUG 61180 | 1.55 |  | 99.56 | 100.00 | 99.50 |  | 96.17 | 100.00 | 95.83 |  | 97.27 | 99.70 | 99.50 |
| SFY-C5 | Ckr CCUG 44504 | 2.078 |  | 99.56 | 100.00 | 99.50 |  | 95.92 | 99.74 | 95.83 |  | 97.33 | 99.79 | 99.50 |
| SFY-C6 | Ckr CCUG 61180 | 1.906 |  | 99.56 | 100.00 | 99.57 |  | 96.13 | 100.00 | 96.04 |  | 97.27 | 99.70 | 99.57 |
| SFY-C7 | Ckr CCUG 61180 | 1.714 |  | 99.56 | 100.00 | 99.57 |  | 96.13 | 100.00 | 96.04 |  | 97.27 | 99.70 | 99.57 |
| SFY-C8 | Ckr CCUG 44504 | 1.884 |  | 99.56 | 100.00 | 99.57 |  | 95.66 | 99.49 | 95.59 |  | 97.07 | 99.49 | 99.57 |
| SFY-C10 | Ckr CCUG 44504 | 1.625 |  | 99.56 | 100.00 | 99.57 |  | 96.08 | 99.74 | 95.50 |  | 96.86 | 99.29 | 99.57 |
| SFY-D5 | Ckr CCUG 61180 | 1.832 |  | 99.56 | 100.00 | 99.50 |  | 96.12 | 100.00 | 95.78 |  | 97.27 | 99.70 | 99.50 |
| SFY-D6 | Ckr CCUG 49276 | 1.664 |  | 99.56 | 100.00 | 99.57 |  | 96.12 | 100.00 | 95.78 |  | 97.17 | 99.49 | 99.57 |
| SFY-D8 | Ckr CCUG 44504 | 1.871 |  | 99.63 | 99.71 | 99.71 |  | 96.12 | 97.42 | 95.78 |  | 97.06 | 99.29 | 99.71 |
| SFY-D10 | Ckr CCUG 44504 | 1.857 |  | 99.56 | 100.00 | 99.50 |  | 96.12 | 100.00 | 96.03 |  | 97.17 | 99.60 | 99.50 |
| SFY-E2 | Ckr CCUG 61180 | 1.711 |  | 99.56 | 100.00 | 99.50 |  | 96.12 | 100.00 | 95.78 |  | 97.37 | 99.80 | 99.50 |
| SFY-E3 | Ckr CCUG 61180 | 1.491 |  | 99.56 | 100.00 | 99.57 |  | 96.12 | 100.00 | 96.03 |  | 97.41 | 99.79 | 99.57 |
| SFY-E5 | Ckr CCUG 61180 | 1.853 |  | 99.56 | 100.00 | 99.50 |  | 96.12 | 100.00 | 96.28 |  | 96.46 | 98.79 | 99.50 |
| SFY-E6 | Ckr CCUG 61180 | 1.786 |  | 99.56 | 100.00 | 99.57 |  | 96.12 | 100.00 | 96.03 |  | 96.66 | 98.99 | 99.57 |
| SFY-E7 | Ckr CCUG 44504 | 1.615 |  | 99.56 | 100.00 | 99.50 |  | 96.12 | 100.00 | 96.03 |  | 97.06 | 99.39 | 99.50 |
| SFY-E10 | Ckr CCUG 44504 | 1.68 |  | 99.56 | 100.00 | 99.57 |  | 96.13 | 100.00 | 96.04 |  | 97.45 | 99.90 | 99.57 |
| SFY-F2 | Ckr CCUG 61180 | 1.67 |  | 99.56 | 100.00 | 99.50 |  | 95.92 | 99.74 | 96.08 |  | 97.47 | 99.90 | 99.50 |
| SFY-F4 | Ckr CCUG 61180 | 1.715 |  | 99.56 | 100.00 | 99.57 |  | 96.12 | 100.00 | 96.28 |  | 96.96 | 99.39 | 99.57 |
| SFY-F5 | Ckr CCUG 61180 | 1.862 |  | 99.56 | 100.00 | 99.50 |  | 96.12 | 100.00 | 96.03 |  | 96.96 | 99.39 | 99.50 |
| SFY-F6 | Ckr CCUG 61180 | 1.712 |  | 99.56 | 100.00 | 99.43 |  | 96.13 | 100.00 | 96.04 |  | 96.87 | 99.29 | 99.43 |
| SFY-F7 | Ckr CCUG 44504 | 2.423 |  | 99.63 | 99.70 | 99.71 |  | 96.12 | 97.42 | 96.03 |  | 96.85 | 98.98 | 99.71 |
| SFY-F8 | Ckr CCUG 61180 | 1.767 |  | 99.56 | 100.00 | 99.50 |  | 96.12 | 100.00 | 96.03 |  | 96.96 | 99.39 | 99.50 |
| SFY-F9 | Ckr CCUG 61180 | 2.016 |  | 99.56 | 100.00 | 99.57 |  | 96.12 | 100.00 | 96.03 |  | 97.27 | 99.70 | 99.57 |
| SFY-F10 | Ckr CCUG 61180 | 1.901 |  | 99.56 | 100.00 | 99.50 |  | 96.13 | 100.00 | 96.04 |  | 97.06 | 99.49 | 99.50 |
| SFY-G1 | Ckr CCUG 44504 | 1.956 |  | 99.56 | 100.00 | 99.50 |  | 96.76 | 100.00 | 95.85 |  | 96.56 | 98.99 | 99.50 |
| SFY-G2 | Ckr CCUG 44504 | 1.694 |  | 99.56 | 100.00 | 99.50 |  | 96.13 | 100.00 | 96.04 |  | 97.47 | 99.90 | 99.50 |
| SFY-G3 | Ckr CCUG 61180 | 2.026 |  | 99.56 | 100.00 | 99.57 |  | 95.88 | 99.74 | 95.54 |  | 96.96 | 99.39 | 99.57 |
| SFY-G5 | Ckr CCUG 61180 | 1.771 |  | 99.56 | 100.00 | 99.50 |  | 96.34 | 100.00 | 96.26 |  | 97.17 | 99.60 | 99.50 |
| SFY-G6 | Ckr CCUG 44504 | 1.938 |  | 99.56 | 100.00 | 99.57 |  | 96.12 | 100.00 | 95.78 |  | 97.47 | 99.90 | 99.57 |
| SFY-G8 | Ckr CCUG 44504 | 1.73 |  | 99.56 | 100.00 | 99.57 |  | 96.12 | 100.00 | 96.03 |  | 97.27 | 99.70 | 99.57 |
| SFY-G9 | Ckr CCUG 49276 | 1.744 |  | 99.56 | 100.00 | 99.57 |  | 96.34 | 100.00 | 95.75 |  | 97.17 | 99.60 | 99.57 |
| SFY-G10 | Ckr CCUG 61180 | 1.668 |  | 99.56 | 100.00 | 99.57 |  | 96.11 | 100.00 | 95.77 |  | 97.17 | 99.60 | 99.57 |
| SFY-H3 | Ckr CCUG 44504 | 1.956 |  | 99.56 | 100.00 | 99.57 |  | 96.13 | 100.00 | 95.80 |  | 97.39 | 99.79 | 99.57 |
| SFY-H4 | Ckr CCUG 61180 | 1.951 |  | 99.56 | 100.00 | 99.57 |  | 95.88 | 99.74 | 95.79 |  | 97.27 | 99.70 | 99.57 |
| SFY-H5 | Ckr DSM 44385T | 1.29 |  | 99.56 | 100.00 | 99.57 |  | 96.11 | 100.00 | 95.78 |  | 97.37 | 99.80 | 99.57 |
| SFY-H6 | Ckr CCUG 61180 | 1.748 |  | 99.56 | 100.00 | 99.57 |  | 96.34 | 100.00 | 96.41 |  | 97.37 | 99.80 | 99.57 |
| SFY-H7 | Ckr CCUG 61180 | 1.902 |  | 99.56 | 100.00 | 99.57 |  | 96.61 | 97.14 | 97.51 |  | 96.84 | 96.74 | 99.57 |
| SFY-H8 | Ckr CCUG 61180 | 2.014 |  | 99.56 | 100.00 | 99.57 |  | 96.54 | 100.00 | 95.92 |  | 97.37 | 99.80 | 99.57 |
| SFY-H9 | Ckr CCUG 61180 | 1.869 |  | 99.56 | 100.00 | 99.57 |  | 96.34 | 100.00 | 95.77 |  | 97.06 | 99.49 | 99.57 |
| SFY-H10 | Ckr CCUG 61180 | 1.773 |  | 99.56 | 100.00 | 99.57 |  | 96.34 | 100.00 | 95.52 |  | 97.37 | 99.80 | 99.57 |
| SFY-I1 | Ckr CCUG 44504 | 1.841 |  | 99.56 | 100.00 | 99.50 |  | 96.77 | 100.00 | 95.61 |  | 97.27 | 99.70 | 99.50 |
| SFY-I2 | Ckr CCUG 61180 | 1.801 |  | 99.56 | 100.00 | 99.50 |  | 96.34 | 100.00 | 96.01 |  | 97.27 | 99.70 | 99.50 |
| SFY-I6 | Ckr CCUG 44504 | 1.764 |  | 99.56 | 100.00 | 99.50 |  | 95.73 | 98.93 | 95.14 |  | 97.17 | 99.60 | 99.50 |
| SFY-I7 | Ckr CCUG 44504 | 1.861 |  | 99.56 | 100.00 | 99.57 |  | 97.79 | 100.00 | 96.70 |  | 97.57 | 100.00 | 99.57 |
| SFY-I8 | Ckr CCUG 61180 | 1.947 |  | 99.56 | 100.00 | 99.50 |  | 96.11 | 100.00 | 96.02 |  | 97.37 | 99.80 | 99.50 |
| SFY-J2 | Ckr CCUG 61180 | 1.708 |  | 99.56 | 100.00 | 99.50 |  | 96.32 | 99.74 | 95.51 |  | 97.47 | 99.90 | 99.50 |
| SFY-J3 | Ckr CCUG 61180 | 1.855 |  | 99.56 | 100.00 | 99.57 |  | 96.46 | 100.00 | 95.79 |  | 97.27 | 99.70 | 99.57 |
| SFY-J4 | Ckr DSM 44385T | 1.546 |  | 99.56 | 100.00 | 99.50 |  | 96.34 | 100.00 | 96.01 |  | 97.27 | 99.70 | 99.50 |
| SFY-J5 | Ckr CCUG 61180 | 1.605 |  | 99.56 | 100.00 | 99.57 |  | 96.34 | 100.00 | 95.75 |  | 97.17 | 99.60 | 99.57 |
| SFY-J7 | Ckr CCUG 44504 | 2.035 |  | 99.56 | 99.63 | 99.64 |  | 96.08 | 97.13 | 95.49 |  | 96.96 | 99.09 | 99.64 |
| SFY-J9 | Ckr CCUG 61180 | 1.836 |  | 99.56 | 100.00 | 99.50 |  | 96.39 | 97.16 | 97.53 |  | 97.13 | 96.93 | 99.5 |
| SFY-J10 | Ckr CCUG 61180 | 1.83 |  | 99.56 | 100.00 | 99.57 |  | 96.34 | 100.00 | 96.01 |  | 97.57 | 100.00 | 99.57 |
| SFY-K1 | Ckr CCUG 44504 | 1.955 |  | 99.56 | 100.00 | 99.57 |  | 96.58 | 100.00 | 96.15 |  | 97.13 | 99.59 | 99.5 |
| SFY-K3 | Ckr CCUG 61180 | 1.397 |  | 99.41 | 99.85 | 99.35 |  | 96.61 | 97.13 | 97.46 |  | 97.16 | 97.06 | 99.35 |
| SFY-K4 | Ckr CCUG 44504 | 1.704 |  | 99.56 | 100.00 | 99.50 |  | 96.34 | 100.00 | 96.41 |  | 97.51 | 99.90 | 99.50 |
| SFY-K5 | Ckr CCUG 44504 | 1.553 |  | 99.56 | 100.00 | 99.57 |  | 96.11 | 100.00 | 95.78 |  | 97.49 | 99.90 | 99.57 |
| SFY-K6 | Ckr CCUG 44504 | 1.914 |  | 99.56 | 100.00 | 99.50 |  | 96.11 | 100.00 | 95.78 |  | 97.48 | 99.89 | 99.50 |
| SFY-K7 | Ckr CCUG 61180 | 1.679 |  | 99.56 | 100.00 | 99.50 |  | 96.34 | 100.00 | 95.75 |  | 97.62 | 100.00 | 99.50 |
| SFY-K8 | Ckr CCUG 61180 | 1.849 |  | 99.56 | 100.00 | 99.50 |  | 96.34 | 100.00 | 96.02 |  | 97.5 | 100.00 | 99.50 |
| SFY-L1 | Ckr CCUG 44504 | 1.738 |  | 99.56 | 100.00 | 99.57 |  | 96.34 | 100.00 | 96.01 |  | 97.61 | 100.00 | 99.57 |
| SFY-L2 | Ckr CCUG 61180 | 1.09 |  | 99.56 | 100.00 | 99.50 |  | 96.61 | 97.13 | 97.25 |  | 97.17 | 97.06 | 99.50 |
| SFY-L3 | Ckr CCUG 61180 | 1.691 |  | 99.56 | 100.00 | 99.57 |  | 96.32 | 99.74 | 95.48 |  | 97.57 | 100.00 | 99.57 |
| SFY-L6 | Ckr CCUG 49276 | 1.281 |  | 99.56 | 100.00 | 99.50 |  | 96.57 | 100.00 | 95.96 |  | 97.58 | 100.00 | 99.50 |
| SFY-M1 | Ckr CCUG 44504 | 1.573 |  | 99.56 | 100.00 | 99.57 |  | 96.54 | 100.00 | 95.42 |  | 96.85 | 99.19 | 99.57 |
| SFY-M2 | Ckr CCUG 61180 | 1.864 |  | 99.56 | 100.00 | 99.43 |  | 96.55 | 100.00 | 95.69 |  | 97.32 | 99.69 | 99.43 |
| SFY-M3 | Ckr CCUG 61180 | 1.481 |  | 99.56 | 100.00 | 99.50 |  | 96.56 | 100.00 | 95.96 |  | 97.50 | 99.90 | 99.50 |
| SFY-M6 | Ckr CCUG 49276 | 1.509 |  | 99.56 | 100.00 | 99.50 |  | 96.55 | 100.00 | 95.43 |  | 97.59 | 100.00 | 99.50 |
| SFY-M9 | Ckr CCUG 61180 | 1.565 |  | 99.56 | 100.00 | 99.57 |  | 96.58 | 100.00 | 95.98 |  | 97.47 | 99.90 | 99.57 |
| SFY-M10 | Ckr CCUG 61180 | 1.684 |  | 99.56 | 100.00 | 99.50 |  | 96.57 | 100.00 | 95.72 |  | 97.48 | 99.89 | 99.50 |
| SFY-N1 | Ckr CCUG 49276 | 1.657 |  | 99.56 | 100.00 | 99.50 |  | 96.58 | 100.00 | 96.34 |  | 97.62 | 100.00 | 99.50 |
| SFY-N5 | Ckr CCUG 61180 | 1.577 |  | 99.56 | 100.00 | 99.57 |  | 96.57 | 100.00 | 95.71 |  | 97.14 | 99.59 | 99.57 |
| SFY-N6 | Ckr CCUG 49276 | 1.474 |  | 99.56 | 100.00 | 99.49 |  | 96.53 | 10000 | 95.66 |  | 97.34 | 99.80 | 99.49 |
| SFY-N9 | Ckr CCUG 44504 | 1.482 |  | 99.56 | 100.00 | 99.57 |  | 96.77 | 100.00 | 95.64 |  | 97.13 | 99.59 | 99.57 |
| SFY-02 | Ckr CCUG 61180 | 1.296 |  | 99.93 | 99.48 | 99.64 |  | 96.54 | 100.00 | 96.59 |  | 97.17 | 99.60 | 99.64 |
| SFY-04 | C.variabile DSM 44702 | 1.98 |  | 99.56 | 100.00 | 99.50 |  | 96.77 | 100.00 | 95.91 |  | 97.13 | 99.59 | 99.50 |
| **Other CKC group** | |  |  |  |  |  |  |  |  |  |  |  |  |  |
| SFY-M4 | Ckr CCUG 61180 | 1.960 |  | 99.56% | 100% | 99.57% |  | 95.30% | 95.85% | 94.29% |  | 95.62% | 95.31% | 95.69% |
| SFY-K9 | Ckr CCUG 61180 | 1.532 |  | 99.56% | 100% | 99.50% |  | 94.91 % | 95.88% | 94.38% |  | 95.55% | 95.24% | 95.33% |

a DSM 44385^T^, the type strain *C*. *kroppenstedtii* DSM 44385^T^, MC-26^T^, the type strain *C*.*parakroppenstedtii* MC-26^T^, MC-17X^T^, the type strain *C*.*pseudokroppenstedtii* MC-17X^T^

bCkr, *C*. *kroppenstedtii*

**Table S3 Rifampicin antimicrobial susceptibility results for 101 clinical isolates.**

| **Strains** | EUCAST  Zone diameter breakpoints (mm) | Rifampicin  Inhibition Zone Diameter (mm) | | Category^a^ |
| --- | --- | --- | --- | --- |
| ***C*. *pseudokroppenstedtii*** group | S≥30，R＜30 | |  |  |
| SFY-A9 |  | | 34 | S |
| SFY-A10 |  | | 30 | S |
| SFY-B5 |  | | 32 | S |
| SFY-B8 |  | | 40 | S |
| SFY-E8 |  | | 40 | S |
| SFY-H2 |  | | 34 | S |
| SFY-I5 |  | | 40 | S |
| SFY-J1 |  | | 35 | S |
| SFY-K10 |  | | 40 | S |
| SFY-L9 |  | | 40 | S |
| SFY-N3 |  | | 32 | S |
| SFY-01 |  | | 30 | S |
| ***C*. *parakroppenstedtii*** group |  | |  |  |
| SFY-A5 |  | | 38 | S |
| SFY-A6 |  | | 35 | S |
| SFY-A7 |  | | 34 | S |
| SFY-A8 |  | | 32 | S |
| SFY-B1 |  | | 30 | S |
| SFY-B2 |  | | 35 | S |
| SFY-B3 |  | | 34 | S |
| SFY-B6 |  | | 46 | S |
| SFY-B9 |  | | 40 | S |
| SFY-B10 |  | | 36 | S |
| SFY-C1 |  | | 36 | S |
| SFY-C2 |  | | 46 | S |
| SFY-C3 |  | | 44 | S |
| SFY-C5 |  | | 42 | S |
| SFY-C6 |  | | 36 | S |
| SFY-C7 |  | | 42 | S |
| SFY-C8 |  | | 46 | S |
| SFY-C10 |  | | 34 | S |
| SFY-D5 |  | | 40 | S |
| SFY-D6 |  | | 42 | S |
| SFY-D8 |  | | 34 | S |
| SFY-D10 |  | | 30 | S |
| SFY-E2 |  | | 38 | S |
| SFY-E3 |  | | 6 | R |
| SFY-E5 |  | | 46 | S |
| SFY-E6 |  | | 46 | S |
| SFY-E7 |  | | 42 | S |
| SFY-E10 |  | | 30 | S |
| SFY-F2 |  | | 36 | S |
| SFY-F4 |  | | 32 | S |
| SFY-F5 |  | | 40 | S |
| SFY-F6 |  | | 37 | S |
| SFY-F7 |  | | 36 | S |
| SFY-F8 |  | | 42 | S |
| SFY-F9 |  | | 40 | S |
| SFY-F10 |  | | 40 | S |
| SFY-G1 |  | | 35 | S |
| SFY-G2 |  | | 36 | S |
| SFY-G3 |  | | 36 | S |
| SFY-G5 |  | | 28 | S |
| SFY-G6 |  | | 36 | S |
| SFY-G8 |  | | 32 | S |
| SFY-G9 |  | | 34 | S |
| SFY-G10 |  | | 40 | S |
| SFY-H3 |  | | 36 | S |
| SFY-H4 |  | | 38 | S |
| SFY-H5 |  | | 36 | S |
| SFY-H6 |  | | 34 | S |
| SFY-H7 |  | | 34 | s |
| SFY-H8 |  | | 38 | S |
| SFY-H9 |  | | 32 | S |
| SFY-H10 |  | | 30 | S |
| SFY-I1 |  | | 30 | S |
| SFY-I2 |  | | 30 | S |
| SFY-I6 |  | | 40 | S |
| SFY-I7 |  | | 35 | S |
| SFY-I8 |  | | 34 | S |
| SFY-J2 |  | | 36 | S |
| SFY-J3 |  | | 34 | S |
| SFY-J4 |  | | 42 | S |
| SFY-J5 |  | | 34 | S |
| SFY-J7 |  | | 40 | S |
| SFY-J9 |  | | 31 | S |
| SFY-J10 |  | | 42 | S |
| SFY-K1 |  | | 35 | S |
| SFY-K3 |  | | 40 | S |
| SFY-K4 |  | | 38 | S |
| SFY-K5 |  | | 42 | S |
| SFY-K6 |  | | 40 | S |
| SFY-K7 |  | | 33 | S |
| SFY-K8 |  | | 34 | S |
| SFY-L1 |  | | 40 | S |
| SFY-L2 |  | | 40 | S |
| SFY-L3 |  | | 22 | I |
| SFY-L6 |  | | 35 | S |
| SFY-M1 |  | | 40 | S |
| SFY-M2 |  | | 42 | S |
| SFY-M3 |  | | 40 | S |
| SFY-M6 |  | | 40 | S |
| SFY-M9 |  | | 36 | S |
| SFY-M10 |  | | 32 | S |
| SFY-N1 |  | | 32 | S |
| SFY-N5 |  | | 26 | S |
| SFY-N6 |  | | 20 | S |
| SFY-N9 |  | | 35 | S |
| SFY-02 |  | | 26 | S |
| SFY-04 |  | | 40 | S |
| **Other CKC** group |  | |  |  |
| SFY-M4 |  | | 40 | S |
| SFY-K9 |  | | 30 | S |

a: R, resistant; S, susceptible；I，intermediate
